# Pretreatment central quality control for craniospinal irradiation in non-metastatic medulloblastoma: First experiences of the German radiotherapy quality control panel in the SIOP PNET5 MB trial

**DOI:** 10.1007/s00066-020-01707-8

**Published:** 2020-11-23

**Authors:** Stefan Dietzsch, Annett Braesigk, Clemens Seidel, Julia Remmele, Ralf Kitzing, Tina Schlender, Martin Mynarek, Dirk Geismar, Karolina Jablonska, Rudolf Schwarz, Montserrat Pazos, Marc Walser, Silke Frick, Kristin Gurtner, Christiane Matuschek, Semi Ben Harrabi, Albrecht Glück, Victor Lewitzki, Karin Dieckmann, Martin Benesch, Nicolas U. Gerber, Stefan Rutkowski, Beate Timmermann, Rolf-Dieter Kortmann

**Affiliations:** 1 Department for Radiation Oncology, University of Leipzig Medical Center, Stephanstr. 9a, 04103 Leipzig, Germany; 2 Departement of Pediatric Hematology and Oncology, University Medical Center Hamburg-Eppendorf, Hamburg, Germany; 3 Clinic for Particle Therapy, West German Proton Therapy Centre, University of Essen, Essen, Germany; 4 Faculty of Medicine, Department of Radiation Oncology, University of Cologne, Cologne, Germany; 5 Department of Radiation Oncology, University Medical Center Hamburg-Eppendorf, Hamburg, Germany; 6 Department of Radiotherapy and Radiation Oncology, Ludwig Maximilian University Munich, Munich, Germany; 7 Center for Protontherapy, Paul Scherrer Institute, Villigen, Switzerland; 8 Department of Radiotherapy and Radiation Oncology, Hospital Bremen Mitte, Bremen, Germany; 9 Department of Radiotherapy and Radiation Oncology, Faculty of Medicine and University Hospital Carl Gustav Carus, Technical University Dresden, Dresden, Germany; 10 Department of Radiation Oncology, Medical Faculty Heinrich Heine University Duesseldorf, Duesseldorf, Germany; 11 Department of Radiation Oncology and Radiotherapy, Heidelberg University Hospital, Heidelberg, Germany; 12 Radiation Oncology, Munich-Schwabing Municipal Hospital, Munich, Germany; 13 Department of Radiotherapy, University of Wuerzburg, Wuerzburg, Germany; 14 Department of Radiotherapy, Medical University of Vienna, Vienna, Austria; 15 Division of Pediatric Hematology/Oncology, Department of Pediatrics and Adolescent Medicine, Medical University of Graz, Graz, Austria; 16 University Children’s Hospital of Zurich, Zurich, Switzerland

**Keywords:** Quality assurance, Deviation, Brain tumor, Pediatric, Review criteria

## Abstract

**Purpose:**

Several studies have demonstrated the negative impact of radiotherapy protocol deviations on tumor control in medulloblastoma. In the SIOP PNET5 MB trial, a pretreatment radiotherapy quality control (RT-QC) program was introduced. A first analysis for patients enrolled in Germany, Switzerland and Austria with focus on types of deviations in the initial plan proposals and review criteria for modern radiation technologies was performed.

**Methods and patients:**

Sixty-nine craniospinal irradiation (CSI) plans were available for detailed analyses. RT-QC was performed according to protocol definitions on dose uniformity. Because of the lack of definitions for high-precision 3D conformal radiotherapy within the protocol, additional criteria for RT-QC on delineation and coverage of clinical target volume (CTV) and planning target volume (PTV) were defined and evaluated.

**Results:**

Target volume (CTV/PTV) deviations occurred in 49.3% of initial CSI plan proposals (33.3% minor, 15.9% major). Dose uniformity deviations were less frequent (43.5%). Modification of the RT plan was recommended in 43.5% of CSI plans. Unacceptable RT plans were predominantly related to incorrect target delineation rather than dose uniformity. Unacceptable plans were negatively correlated to the number of enrolled patients per institution with a cutoff of 5 patients (*p* = 0.001).

**Conclusion:**

This prospective pretreatment individual case review study revealed a high rate of deviations and emphasizes the strong need of pretreatment RT-QC in clinical trials for medulloblastoma. Furthermore, the experiences point out the necessity of new RT-QC criteria for high-precision CSI techniques.

**Electronic supplementary material:**

The online version of this article (10.1007/s00066-020-01707-8) contains supplementary material, which is available to authorized users.

## Introduction

In standard risk (SR) medulloblastoma, craniospinal irradiation (CSI) followed by a tumor bed boost is considered the standard treatment in non-infant age groups. Retrospective reports showed that inadequate treatment fields had a negative impact on tumor control and outcome [1–3]. This was confirmed in similar clinical settings [4–6]. Thus, there is a broad consensus that pretreatment quality control of radiotherapy plans (RT-QC) is an important measure in multi-institutional clinical trials [7–10].

In 2014, the European branch of the International Society of Pediatric Oncology (SIOPe) initiated the SIOP PNET5 MB trial for children with non-metastatic medulloblastoma with a low-risk or average-risk biological profile (ClinicalTrials.gov identifier: NCT02066220). The quality assurance (QA) program included a central review of pathology, magnetic resonance imaging (MRI), and radiotherapy (RT). Furthermore, pretreatment RT-QC was considered a mandatory component of the study. This report describes a first analysis for patients enrolled in Germany, Austria, and Switzerland, with focus on the type of deviations in the initial plan proposals and review criteria for modern radiation technologies.

## Materials and methods

SIOP PNET5 MB is a prospective phase II/III study in patients between the ages of 3 to 5 years and 21 years with clinical SR medulloblastomas according to the risk group definitions which have been used so far, e.g., in HIT-SIOP PNET4 [11–13]. Patients with low-risk biological profile (PNET5 MB, LR arm) received 18 Gy to the craniospinal axis. All other SR patients (PNET5 MB, SR arm) received 23.4 Gy CSI. The tumor site was boosted up to 54 Gy. Patients recruited to the SIOP PNET5 MB SR and LR strata until December 31, 2018 in Germany, Switzerland and Austria and with complete available DICOM-RT data of CSI plans were considered eligible for this report.

**Fig. 1:**
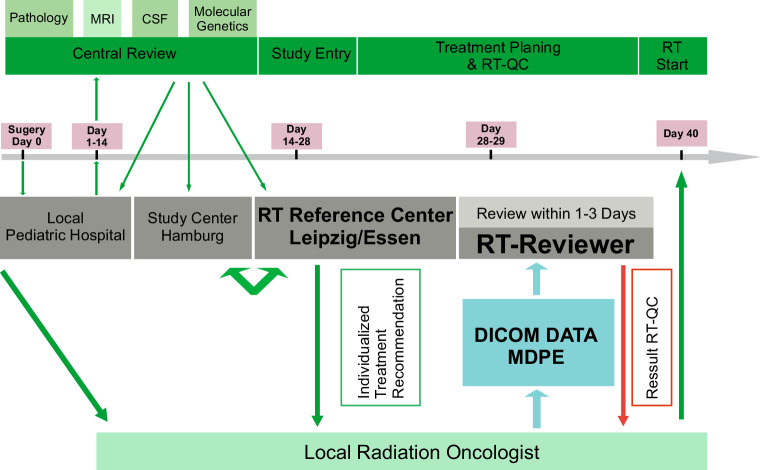
Workflow and time schedule for quality control from surgery to start of radiotherapy. *MRI* Magnetic resonance imaging, *CSF* cerebrospinal fluid, *RT* radiotherapy, *RT-QC* quality control of radiotherapy

RT-QC was performed by the Reference Center for Radiotherapy in Childhood Brain Tumors in Leipzig in collaboration with the West German Proton Center in Essen. Workflow and time schedule of RT-QC are shown in Fig. 1. A benchmark case or dummy run was not performed. All local radiation oncologists received a detailed individualized treatment recommendation including target delineation and dosimetric aims. Central plan analyses were performed by importing the original plan data including dose files into the local treatment planning systems of the reference centers (RayStation, Raysearch Laboratories, Stockholm, Sweden). CSI techniques were documented and grouped into three categories: 3D conformal (lateral opposing fields for brain and posterior fields for spine), high precision photon (intensity-modulated radiotherapy with fixed gantry angles [IMRT], volumetric modulated arc therapy [VMAT], tomotherapy), or proton beam therapy (with active scanning). Typical dose distributions are available in the supplementary material (Fig. S1). In order to analyze possible changes during accrual, time of enrollment was divided in two periods. Cut-off was the median of enrolled patients at the end of 2016.

For SIOP PNET5 MB RT-QC, minor and major deviations of dose uniformity were defined according to ICRU 50/62 (Table 1; [14, 15]). Target deviations were used according to Carrie et al. [1]. These definitions address the distance from the field edge to the clinical target volume (CTV) and are therefore only suitable for simulation-based treatment planning and 3D conformal techniques with opposing lateral fields of the brain and posterior fields of the spinal axis. For high-precision CSI techniques, RT-QC adaptations of the former concept described by Carrie et al. were performed in order to better evaluate the CTV/PTV and their coverage by the 95% isodose, with a special focus on the critical areas cribriform plate, temporal lobe, and thecal sac (Table 1; [1]). Since 2018, CTV has been evaluated according to the SIOP guideline on craniospinal target volume delineation with additional special focus on skull base foramina [16]. The SIOP PNET5 MB protocol required contouring of organs at risk (OAR) and collection of corresponding dose exposure. 30 Gy to at least one cochlea is the only dose constraint to an OAR. Contouring and dose to OAR was not a criterion for plan acceptance.

**Table 1:** SIOP PNET5 protocol definitions of radiotherapy parameters for quality control and additional definitions of target delineation as minor or major deviation of the reference center

	Per protocol	Minor deviation	Major deviation
**Protocol definitions**			
*Target volume*			
CSI (distance from field edge to CTV)			
Cribriform plate	≥5 mm	3 to <5 mm	<3 mm
All other regions	≥10 mm	5 to <10 mm	<5 mm
*Dose uniformity*			
V95%	≥95%	≥90 to <95%	<90%
V107%	≤5%	>5% to <10%	≥10%
**Additional definitions by the reference center**			
*Target volume delineation*			
CTV/PTV	Entire subarachnoid space encompassed by PTV and CTV	Entire subarachnoid space not encompassed by CTV but by PTV	Entire subarachnoid space not encompassed by CTV and PTV

*CSI* Craniospinal irradiation, *CTV* clinical target volume, *PTV* planning target volume, *V95%* proportion of PTV which is covered by the 95% isodose, *V107%* proportion of PTV which receive ≥107% of the prescribed dose

Furthermore, deviations were checked for clinical relevance (expected increased risk for relapse and/or increased risk for toxicity) and scored as “acceptable” or “unacceptable” at the discretion of the reviewer and based on the experience of treatment plan evaluation in the SIOP Neuroblastoma protocol [17]. The overall QA result was rated as “per protocol,” “acceptable deviation,” or “unacceptable deviation.” If modifications were required, results of RT-QC were communicated to the treating institution by telephone and/or Email including illustrating screenshots. The final result was subsequently communicated by a formal letter.

Associations between variables were examined using χ^2^ tests. All statistical analyses were performed using the Statistical Package for Social Sciences (IBM SPSS statistics), version 24 (IBM, Armonk, NY, USA).

## Results

Between September 2014 and December 2018, 70 German, 6 Swiss, and 2 Austrian patients were enrolled in the SIOP PNET5-MB trial and treated in 29 institutions. In 8 patients, no DICOM-RT data were available. One patient was treated by a simulation-based treatment technique. In this patient, modification of RT portals at the cribriform plate was recommended. The patient was excluded from analysis because of the lack of CTV/PTV structures and dose uniformity data. Sixty-nine CSI plans were available for detailed evaluation. Forty-six patients (66.6%) were treated in the SR arm and 23 patients (33.3%) in the LR arm. Supplementary Table 1S summarizes the frequency of different CSI techniques over the evaluated period. The data indicate a shift from 3D conformal techniques being the most common between 2014 and 2016, to proton therapy being the most common to date.

Supplementary Table S2 summarizes the frequency of target volume and dose uniformity deviations in the initial plan proposals. Target volume deviations occurred in 49.3% of RT plans and were more frequent in the brain CTV/PTV (30.4%) than in the spinal CTV/PTV (24.6%). Dose uniformity deviations were found in 43.5% of the RT plans and were more frequent in the spinal PTV. The frequency of dose uniformity deviation was higher for 3D conformal radiotherapy (72.7% major, 22.7% minor) than for high-precision techniques, in which 80.9% were per protocol (χ^2^ *p* < 0.001). Only 1 of 22 3D conformal radiotherapy plans met the dose uniformity criteria of the protocol. For high-precision techniques, the median V95 (proportion of PTV which is covered by the 95% isodose) was 99.1% ± 2% for PTV-brain and 98.3% ± 5.2% for PTV-spine. V107 (proportion of PTV which receives ≥107% of the prescribed dose) was median 0% for both PTVs.

**Fig. 2:**
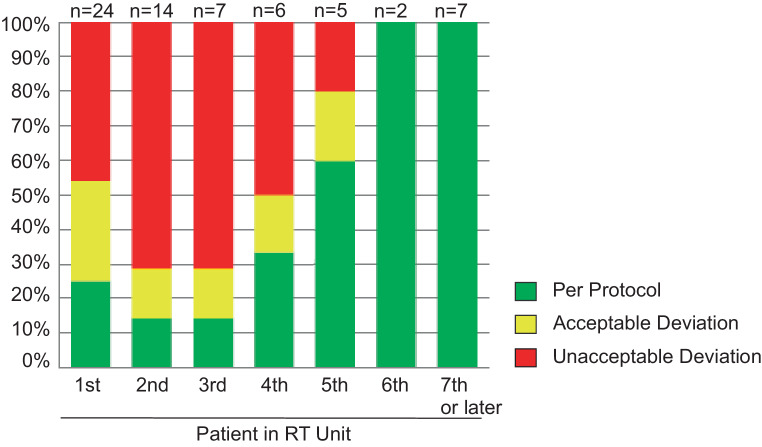
Influence of experience with protocol on quality control result. *n* depicts number of patients who were evaluated with respect to the patients in radiotherapy unit (e.g., 24 patients were evaluated as the first patient in an unit). *RT* Radiotherapy

Only 27 plans (39.1%) did not have any deviation. In 17.4% (*n* = 12) of the RT plans, deviations were considered as acceptable and in 30 (43.5%) plan modifications were recommended. The rate of recommended CSI plan modifications was higher in low-recruiting (≤4 patients) than in high-recruiting (≥5 patients) radiotherapy units (62.1% vs. 30%; χ^2^ *p* = 0.005). An impact of institutional experience in treating SIOP PNET5 MB trial patients on the rate of unaccepted RT plans was also observed (Fig. 2). High rates of unacceptable deviations were seen in the first 4 patients in all RT units. The rate declined when enrolling the fifth and following patients (first to fourth patient in unit 56.9% vs. fifth or later patient in unit 5.6%; χ^2^ *p* < 0.001). The percentage of recommended CSI plan modifications did not decrease over time and was 44.1% in 2014–2016 and 42.9% in 2017–2018 (χ^2^ *p* = 0.916).

Table 2 shows the interaction between the type of deviation and requirement of RT plan modification. Only 1 RT plan with dose uniformity deviation alone required plan modification. In 9 of 30 (30%) cases with unacceptable deviations, only target volume deviations were present, with a correct assessment of dose uniformity. 20 of 30 unacceptable plans showed both types of deviation.

**Table 2:** Interaction between target and dose uniformity deviations with respect to overall result (acceptable/unacceptable) of the initial plan proposals of the local radiation oncologist

	Per protocol	Acceptable deviation	Unacceptable deviation	Total
Correct target and dose	27	0	0	*27*
Correct target but deviation dose uniformity	0	7 dose minor 3 dose major 4	1 dose minor 1	*8*
Deviation target but correct dose uniformity	0	3 target minor 3	9 target minor 6 target major 3	*12*
Deviation target and dose uniformity	0	2 target minor 2 dose major 2	20 target minor 12 target major 8 dose minor 5 dose major 15	*22*
*Total*	*27*	*12*	*30*	*69*

## Discussion

The present study represents a large cohort of a pretreatment, fully digital individual case review RT-QC procedure in medulloblastomas treated by CSI.

### Observed deviations

We observed a high rate of RT protocol deviations in the initial plan proposals (49.3% target delineation, 43.5% dose uniformity). 30 of 69 (43.5%) RT plans were clinically unacceptable and modifications were recommended. High rates of RT protocol deviations were also reported in other medulloblastoma trials. Most of these retrospective studies were based on the evaluation of simulation films of lateral opposing fields for the brain and posterior fields for the spinal canal [1, 2, 18, 19]. First experiences of pretreatment central RT-QC of SIOP PNET5 MB patients treated in Italy revealed a necessity of plan revision in 62.5% of CSI plans. Most common reasons were target delineation and covering of the cribriform plate or the thecal sac [20].

### CTV/PTV delineation

Definitions of targeting deviations in the SIOP PNET5 MB protocol adopted the QC process of the HIT-SIOP PNET4 trial or Carrie et al., respectively [1, 12]. This included the distance from the field edge and bony reference structures to the CTV in lateral opposing fields for the brain and dorsal fields for the spinal canal. The use of this technique, however, decreased from approximately 50% in 2014–2016 to 14% in 2017–2018. Modern high-precision technologies (IMRT, VMAT, tomotherapy, and in particular proton beam therapy) were increasingly used to decrease the risk of late toxicity and can be considered as standard of care today [21–24]. Presently, there is no established standardized definition of CSI targeting deviations for high-precision CSI techniques available. However, both review of target delineation and assessment of dose uniformity are indispensable for evaluating a treatment plan for any risk of relapse. This necessity is emphasized by our findings that target deviations were more common than dose uniformity deviations in the 30 plans considered as “unacceptable” (3.3% unacceptable dose uniformity alone, 30% unacceptable target definition alone, 66.7% both deviations). Similar results were observed in the Italian cohort [20]. Moreover, 9 of our 69 plans (13%) or 9 of 30 unacceptable deviations (30%) would have been scored as acceptable based on the dose uniformity criteria alone defined in the protocol.

#### Dose uniformity and regional dose distribution

Evaluation of dose uniformity deviations has three short comings. In case of posterior treatment fields, major deviations can occur due to a formal underdosage in anterior parts or overdosage in posterior parts of the spinal PTV. This can especially be the case when the whole vertebral bodies are included into the spinal PTV to prevent a dose gradient >5 Gy or >70% within the vertebral bodies [25]. These deviations, when caused by radiation techniques but not due to planning inadequacies, were judged as acceptable deviations not requiring any modification.

**Fig. 3:**
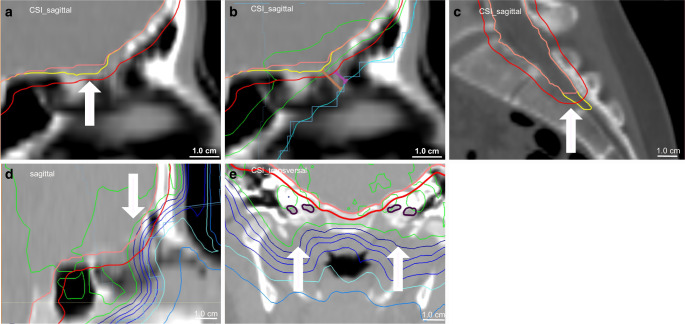
Examples of proposed deviations. **a** Acceptable deviation—the subarachnoid space according SIOP guideline (*yellow*)[16] is not encompassed by CTV (*pink*) but by the PTV (*red*). **b** Background for acceptable deviation in (**a**)—the distance from field edge (*leaf*) and 50% isodose (*light blue*, as equivalent to a customized block) to the PTV (*red*) is 5 mm (*purple*) and to the subarachnoid space (correct reference CTV; *yellow*) approximately 8 mm (*brown*), which is per protocol according to the SIOP PNET5 protocol definitions in Table 1. The 95% isodose does not completely cover the PTV (*red*) but the subarachnoid space (*yellow*). **c** Major/unacceptable deviation—the subarachnoid space (thecal sac) is not encompassed by CTV (*pink*) and PTV (*red*). **d** Unacceptable circumscribed regional dose in case of correct target definition and dose uniformity—the subarachnoid space (cribriform plate) is encompassed by CTV (*pink*) and PTV (*red*) but the 95% isodose does not cover this critical region because of eye-sparing plan optimization. **e** Acceptable plan in case of unacceptable target volume deviation—the subarachnoid space (scull base foramina; *violet*) is not encompassed by CTV (*pink*) and PTV (*red*) but is covered by the 95% isodose (*outer light green line*) due to radiation technique. *SIOP* International Society of Paediatric Oncology, *CTV* clinical target volume, *PTV* planning target volume, *CSI* craniospinal irradiation

The V95% constraint has to be used with caution in the case of large PTVs. The volume of PTV-brain varies depending on age and is more than 1000 cm^3^. The constraint V95% ≥ 95% for a PTV of 1000 cm^3^ means that underdosages in 50 cm^3^ will still be considered as per protocol. The cribriform plate has been demonstrated to be a critical region for relapse in medulloblastoma [1, 3, 26]. Because of the small volume circumscribed, marked underdosage can occur, although formally, a correct dose uniformity was calculated. Therefore, regional dose distribution has to be evaluated, especially in critical regions like the cribriform plate or temporal lobes (Fig. 3).

Furthermore, the circumscribed regional dose distribution should be evaluated in regions of inadequate CTV and PTV delineation. In our cohort, five cases of inadequate CTV were identified not including the skull base foramina. But for technical reasons, this region was encompassed by the 95% isodose. Therefore, this major targeting deviation was judged as acceptable without requiring any modification (Fig. 3).

As a consequence, major deviations were not automatically unacceptable. On the other hand, a plan with formally correct dose uniformity could be unacceptable, e.g., because of inadequate CTV delineation or dose coverage at critical regions. Due to these observations, it seems to be necessary to renew the criteria for CSI-QC and to define acceptable and unacceptable deviations.

### New QC criteria

From our experience, we would propose defining different criteria for 3D conformal plans (lateral opposing fields for brain and posterior fields for spine) and high-precision CSI techniques. QC of 3D conformal plans can be based on the criteria of Carrie et al. [1]. The dose uniformity criteria can be used for PTV-brain but seem to be unsuitable for the spinal PTV when covered by posterior spinal fields. For high-precision techniques, we propose the new criteria shown in Table 3 and illustrated in Fig. 3, which include CTV/PTV contouring, circumscribed regional dose, vertebral dose in children before the end of the pubertal acceleration phase [25], and dose uniformity. The basis of RT-QC criteria is dose coverage of the entire subarachnoid space defined according to the SIOP contouring guideline [16]. CTV delineation according to this guideline is strongly recommended. The criteria for CTV/PTV delineation adopt the margins between field edge and CTV of Carrie et al. [1] and the SIOP PNET 4 protocol [12]. Due to the typical lateral dose gradient in photon therapy, approximately 50% of the prescribed dose is given at the field edge and 95% of dose is applied approximately 3 to 5 mm distant to the field edge within the target. Furthermore, we find adequate dose uniformity in the majority of cases when high-precision CSI techniques were used. Therefore, we propose using a stricter dose uniformity constraint (V95% ≥ 98%) as was recommended by the ICRU 83 report for IMRT techniques [27]. The same constraints should also be used for proton plans, respecting the recommendations of the ICRU 78 report [28].

**Table 3:** Proposal for definitions of acceptable and unacceptable deviations and final result of quality control in craniospinal irradiation with high-precision techniques

	Acceptable deviation	Unacceptable deviation
**Target volume delineation**		
*CTV/PTV*	Entire subarachnoid space^a^ *not encompassed by CTV* but by PTV or PTV larger than necessary (≤0.5 cm areas with effect on OAR dose^b^ ≤1.0 cm areas without effect on OAR dose^b^)	Entire subarachnoid space^a^ *not encompassed by CTV and PTV* or PTV substantially larger than necessary (>0.5 cm areas with effect on OAR dose^b^ >1.0 cm areas without effect on OAR dose^b^)
**Circumscribed regional dose coverage at critical locations (cribriform plate, temporal lobes, skull base foramina, thecal sac)**		
*95%-isodose*	Not defined	Does not encompass entire subarachnoid space^a^
**Vertebral dose in children before end of pubertal acceleration phase**		
*Anterior-posterior dose gradient*	Not defined	>5 Gy or 70%^c^
**Dose uniformity**		
*V95%*	≥95 to <98%	<95%
*V107%*	>5% to <10%	≥10%
**Final result of RT-QC**		
*Per protocol*		
*Acceptable deviation* *(in individual cases, when missing part of target volume is covered by dose due to radiation technique, final result can be acceptable deviation even in case of unacceptable target volume deviation)*		
*Unacceptable deviation*		

*CTV* Clinical target volume, *PTV* planning target volume, *OAR* organ at risk, *V95%* proportion of PTV which is covered by the 95% isodose, *V107%* proportion of PTV which receive ≥107% of the prescribed dose, *RT-QC* quality control of radiotherapy, *SIOP* International Society of Paediatric Oncology

^a^Correct reference CTV according SIOP guideline [16]

^b^For example, eye globe, lens, thyroid, lung, heart, esophagus, kidney

^c^according SIOP consensus recommendations [25]

### Impact of experience with RT guidelines of the protocol

The rate of unaccepted RT plans in our cohort depends on the experience of the radiotherapy units in treating SIOP PNET5 MB patients, with a cutoff of 5 patients per institution. A comparable impact on the institution’s familiarity with the protocol was seen in RT-QC of meningioma in the EORTC 22042-26042 trial [6]. Fewer deviations were observed in RT plans from high-recruiting institutions (≥5 patients) compared to those from low-recruiting centers (22% vs. 62%, *p* = 0.007). The recently published Italian experience of SIOP PNET5 MB RT-QC also showed a higher rate of corrected major deviations in patients when treated in less experienced centers (88.2%) compared to the whole cohort (62.5%) [20]. Interestingly, a decrease in unacceptable RT plans over time was not observed. This could partly be explained by the continuous high percentage of patients included by low-recruiting radiotherapy units (≤4 patients; 47.1% in 2014–2016 and 37.1% in 2017–2018; *p* = 0.404). Additionally, the number of new institutions starting recruitment of patients (patient 1 to 4) remained high in the second period (57.1%).

Our observations underline the fundamental role of up-front RT-QC to ensure protocol-compliant RT and confirmed the findings of the M‑SFOP 98 protocol. In this trial, craniospinal fields were reviewed before the start of radiotherapy. Major deviations were observed in 14 of 55 patients; 9 of these deviations were due to eye shielding and 8 of them were modified before RT start [29]. It remains to be seen how far the recently published European guideline for craniospinal CTV contouring will help to improve target delineation [16]. Furthermore, a training program for local radiation oncologists including benchmark cases and with a final certification of investigators has to be considered.

## Conclusion

This pretreatment individual case review study revealed a high rate of protocol deviations and emphasizes the strong need for pretreatment RT-QC in clinical trials on medulloblastoma, particularly in low-recruiting centers and for approximately the first 5 patients of each RT institution. Therefore, pretreatment QA programs are desirable to support decentralized treatment in multicenter trials. Moreover, our experiences point out the necessity of new RT-QC criteria for high-precision CSI techniques.

## Caption Electronic Supplementary Material

Supplementary Fig. 1 Typical dose distributions of CSI techniques

Supplementary Table 1 CSI techniques

Supplementary Table 2 Deviation of target volume delineation and dose uniformity. ^a^Classification of deviations based on the definitions in table 1B. ^b^Number of minor deviations in total is lower than the single items because of possible combination with a major deviation of another item leading to a major total result

## References

[1] Carrie C, Hoffstetter S, Gomez F (1999). Impact of targeting deviations on outcome in medulloblastoma: study of the French Society of Pediatric Oncology (SFOP). Int J Radiat Oncol Biol Phys.

[2] Kortmann RD, Timmermann B, Kuhl J (1999). HIT ’91 (prospective, co-operative study for the treatment of malignant brain tumors in childhood): accuracy and acute toxicity of the irradiation of the craniospinal axis. Results of the quality assurance program. Strahlenther Onkol.

[3] Miralbell R, Bleher A, Huguenin P (1997). Pediatric medulloblastoma: radiation treatment technique and patterns of failure. Int J Radiat Oncol Biol Phys.

[4] Fairchild A, Weber DC, Bar-Deroma R (2012). Quality assurance in the EORTC 22033-26033/CE5 phase III randomized trial for low grade glioma: the digital individual case review. Radiother Oncol.

[5] Gondi V, Cui Y, Mehta MP (2015). Real-time pretreatment review limits unacceptable deviations on a cooperative group radiation therapy technique trial: quality assurance results of RTOG 0933. Int J Radiat Oncol Biol Phys.

[6] Coskun M, Straube W, Hurkmans CW (2013). Quality assurance of radiotherapy in the ongoing EORTC 22042-26042 trial for atypical and malignant meningioma: results from the dummy runs and prospective individual case reviews. Radiat Oncol.

[7] Goodman KA (2013). Quality assurance for radiotherapy: a priority for clinical trials. JNCI J Natl Cancer Inst.

[8] Weber DC, Tomsej M, Melidis C (2012). QA makes a clinical trial stronger: evidence-based medicine in radiation therapy. Radiother Oncol.

[9] FitzGerald TJ (2014). A new model for imaging and radiation therapy quality assurance in the National Clinical Trials Network of the National Cancer Institute. Int J Radiat Oncol Biol Phys.

[10] Fairchild A, Straube W, Laurie F (2013). Does quality of radiation therapy predict outcomes of multicenter cooperative group trials? A literature review. Int J Radiat Oncol Biol Phys.

[11] Bartlett F, Kortmann R, Saran F (2013). Medulloblastoma. Clin Oncol.

[12] Lannering B, Rutkowski S, Doz F (2012). Hyperfractionated versus conventional radiotherapy followed by chemotherapy in standard-risk medulloblastoma: results from the randomized multicenter HIT-SIOP PNET 4 trial. J Clin Oncol.

[13] Zeltzer PM, Boyett JM, Finlay JL (1999). Metastasis stage, adjuvant treatment, and residual tumor are prognostic factors for medulloblastoma in children: conclusions from the Children’s Cancer Group 921 randomized phase III study. J Clin Oncol.

[14] Landberg T, Chavaudra J, Dobbs J (2016). Report 50. J ICRU.

[15] Landberg T, Chavaudra J, Dobbs J (2016). Report 62. J ICRU.

[16] Ajithkumar T, Horan G, Padovani L (2018). SIOPE—brain tumor group consensus guideline on craniospinal target volume delineation for high-precision radiotherapy. Radiother Oncol.

[17] Gaze MN, Boterberg T, Dieckmann K (2013). Results of a quality assurance review of external beam radiation therapy in the International Society of Paediatric Oncology (Europe) Neuroblastoma Group’s high-risk neuroblastoma trial: a SIOPEN study. Int J Radiat Oncol Biol Phys.

[18] Miralbell R, Fitzgerald TJ, Laurie F (2006). Radiotherapy in pediatric medulloblastoma: quality assessment of Pediatric Oncology Group trial 9031. Int J Radiat Oncol Biol Phys.

[19] Donahue B, Marymont MAH, Kessel S (2012). Radiation therapy quality in CCG/POG intergroup 9961: implications for craniospinal irradiation and the posterior fossa boost in future medulloblastoma trials. Front Oncol.

[20] Meroni S, Cavatorta C, Barra S (2019). Ein spezielles Cloud-System für die Real-time‑/Vorab-Qualitätssicherung in der pädiatrischen Strahlentherapie. Strahlenther Onkol.

[21] Laprie A, Hu Y, Alapetite C (2015). Paediatric brain tumours: a review of radiotherapy, state of the art and challenges for the future regarding protontherapy and carbontherapy. Cancer Radiother.

[22] Yock TI, Yeap BY, Ebb DH (2016). Long-term toxic effects of proton radiotherapy for paediatric medulloblastoma: a phase 2 single-arm study. Lancet Oncol.

[23] Eaton BR, Esiashvili N, Kim S (2016). Endocrine outcomes with proton and photon radiotherapy for standard risk medulloblastoma. Neuro Oncol.

[24] Huynh M, Marcu LG, Giles E (2019). Are further studies needed to justify the use of proton therapy for paediatric cancers of the central nervous system? A review of current evidence. Radiother Oncol.

[25] Hoeben BA, Carrie C, Timmermann B (2019). Management of vertebral radiotherapy dose in paediatric patients with cancer: consensus recommendations from the SIOPE radiotherapy working group. Lancet Oncol.

[26] Taylor RE, Donachie PHJ, Weston CL (2009). Impact of radiotherapy parameters on outcome for patients with supratentorial primitive neuro-ectodermal tumours entered into the SIOP/UKCCSG PNET 3 study. Radiother Oncol.

[27] The International Commission on Radiation Units and Measurements (2010). Prescribing, recording, and reporting intensity-modulated photon-beam therapy (IMRT). J ICRU.

[28] The International Commission on Radiation Units and Measurements (2007). Prescribing, recording and reporting proton-beam therapy. J ICRU.

[29] Carrie C, Muracciole X, Gomez F (2005). Conformal radiotherapy, reduced boost volume, hyperfractionated radiotherapy, and online quality control in standard-risk medulloblastoma without chemotherapy: results of the French M-SFOP 98 protocol. Int J Radiat Oncol Biol Phys.

