# Supplementary material for: Pretreatment central quality control for craniospinal irradiation in non-metastatic medulloblastoma: First experiences of the German radiotherapy quality control panel in the SIOP PNET5 MB trial
Source: Strahlenther Onkol. 2020 Nov 23;197(8):674–82. doi: 10.1007/s00066-020-01707-8 (PMC8292275; doi:10.1007/s00066-020-01707-8)

Supplementary figure 1

Typical dose distributions of CSI techniques

3d conformal

lateral opposing fields for brain and posterior fields for spine

high precision photon

volumetric modulated arc therapy [VMAT]

proton beam therapy

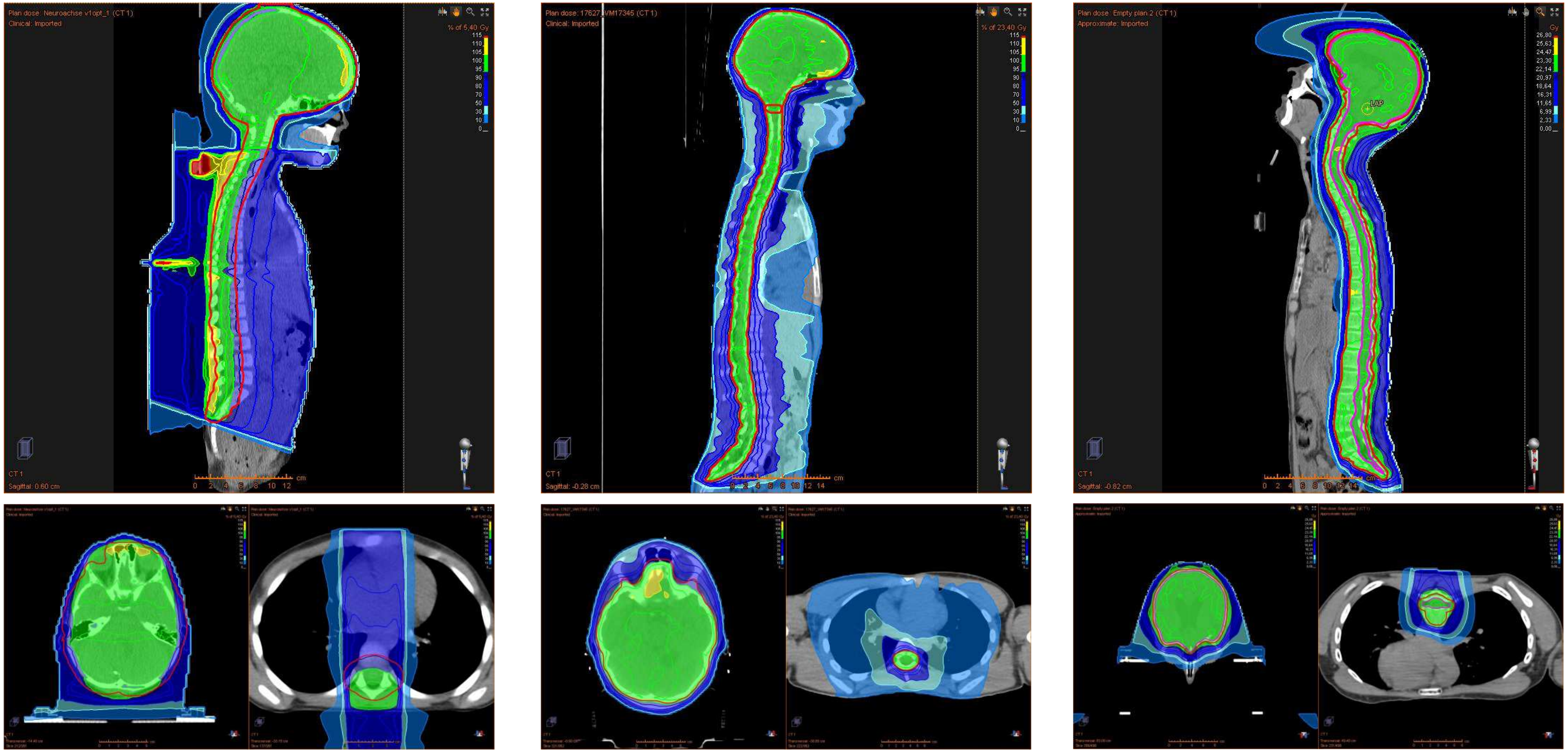

Supplement: Supplementary file 1 — Supplementary Fig. 1 Typical dose distributions of CSI techniques [file 66_2020_1707_MOESM1_ESM.pdf]
