# Supplementary material for: Pretreatment central quality control for craniospinal irradiation in non-metastatic medulloblastoma: First experiences of the German radiotherapy quality control panel in the SIOP PNET5 MB trial
Source: Strahlenther Onkol. 2020 Nov 23;197(8):674–82. doi: 10.1007/s00066-020-01707-8 (PMC8292275; doi:10.1007/s00066-020-01707-8)
Supplement: Supplementary file 2 — Supplementary Table 1 CSI techniques [file 66_2020_1707_MOESM2_ESM.docx]

|  | Whole time period  2014-2018(n=69) | Time period  2014-2016  (n=34) | Time period  2017-2018  (n=35) |
| --- | --- | --- | --- |
| 3D conformal | 22 (31.9%) | 17 (50.0%) | 5 (14.3%) |
| High precision photon  (IMRT, VMAT, tomotherapy) | 21 (30.4%) | 9 (26.5%) | 12 (34.3%) |
| Proton beam therapy | 26 (37.7%) | 8 (23.5%) | 18 (51.4%) |

Supplemantary table 1: CSI techniques
