# Supplementary material for: Pretreatment central quality control for craniospinal irradiation in non-metastatic medulloblastoma: First experiences of the German radiotherapy quality control panel in the SIOP PNET5 MB trial
Source: Strahlenther Onkol. 2020 Nov 23;197(8):674–82. doi: 10.1007/s00066-020-01707-8 (PMC8292275; doi:10.1007/s00066-020-01707-8)
Supplement: Supplementary file 3 — Supplementary Table 2 Deviation of target volume delineation and dose uniformity. aClassification of deviations based on the definitions in table 1B. bNumber of minor deviations in total is lower than the single items because of possible combination with a major deviation of another item leading to a major total result [file 66_2020_1707_MOESM3_ESM.docx]

|  | Minor deviation | Major deviation | Total deviation |
| --- | --- | --- | --- |
| Target volume total^a)^ | 23 (33.3%) | 11 (15.9%) | **34 (49.3%)** |
| Target volume spinal ^a)^ | 11 (15.9%) | 6 (8.7%) | 17 (24.6%) |
| Target volume brain ^a)^ | 15 (21.7%) | 6 (8.7%) | 21 (30.4%) |
| Target volume and circumscribed regional dose | | |  |
| Cribriform plate | 12 (17.4%) | 6 (8.7%) | 18 (26.1%) |
| Temporal lobe | 4 (5.8%) | 4 (5.8%) | 8 (11.6%) |
| Dose uniformity total | 9 (13%)^b)^ | 21 (30.4%) | **30 (43.5%)** |
| V95% brain | 9 (13%)^b)^ | 5 (7.2%) | 14 (20.2%) |
| V107% brain | 0 | 1 (1.4%) | 1 (1.4%) |
| V95% spinal | 12 (17.4%)^b)^ | 15 (21.7%) | 27 (39.1%) |
| V107% spinal | 4 (5.8%)^b)^ | 8 (11.6%) | 12 (17.4%) |

Supplementary table 2: Deviation of target volume delineation and dose uniformity.

^a)^ Classification of deviations based on the definitions in table 1B

^b)^ Number of minor deviation in total is lower than the single items because of possible combination with a major deviation of another item leading to a major total result
